# Lesions of either anterior orbitofrontal cortex or ventrolateral prefrontal cortex in marmoset monkeys heighten innate fear and attenuate active coping behaviors to predator threat

**DOI:** 10.3389/fnsys.2014.00250

**Published:** 2015-01-21

**Authors:** Yoshiro Shiba, Charissa Kim, Andrea M. Santangelo, Angela C. Roberts

**Affiliations:** ^1^Department of Physiology, Development and Neuroscience, University of CambridgeCambridge, UK; ^2^Behavioural and Clinical Neuroscience Institute, University of CambridgeCambridge, UK

**Keywords:** anxiety, emotion regulation, primate, prefrontal cortex, snake fear

## Abstract

The ventral prefrontal cortex is an integral part of the neural circuitry that is dysregulated in mood and anxiety disorders. However, the contribution of its distinct sub-regions to the regulation of negative emotion are poorly understood. Recently we implicated both the ventrolateral prefrontal cortex (vlPFC) and anterior orbitofrontal cortex (antOFC) in the regulation of conditioned fear and anxiety responses to a social stimulus, i.e., human intruder, in the marmoset monkey. In the present study we extend our investigations to determine the role of these two regions in regulating innate responses and coping strategies to a predator stimulus, i.e., a model snake. Both the vlPFC and antOFC lesioned groups exhibited enhanced anxiety-related responses to the snake in comparison to controls. Both groups also showed a reduction in active coping behavior. These results indicate that the vlPFC and antOFC contribute independently to the regulation of both innate fear and, as previously reported, conditioned fear, and highlight the importance of these regions in producing stimulus-appropriate coping responses. The finding that dysregulation in two distinct prefrontal regions produces the apparently similar behavioral phenotype of heightened negative emotion provides insight into the varied etiology that may underlie this symptom across a wide variety of neuropsychiatric conditions with implications for personalized treatment strategies.

## 1. Introduction

Ever since the 19th century case report of Phineas Gage, whose emotional character dramatically changed after considerable damage to his ventromedial prefrontal cortex, the PFC has been the focus of investigation for the regulation of emotions. Although negative emotions such as anxiety and fear are adaptive responses, the appropriate regulation of such negative emotions is crucial for a healthy mental life. When dysregulated, excessive fear and anxiety can become maladaptive and interfere with one's personal and social well-being. Recent studies using brain-imaging technologies have reported abnormal activities within the prefrontal areas of patients suffering from such disruptive anxiety disorders. When exposed to fear-inducing stimuli such as phobic objects (e.g., snake, spider etc.), patients with posttraumatic stress disorder (PTSD), panic disorder and specific phobia exhibit reduced ventromedial PFC activity (Etkin et al., 2007; Killgore et al., 2013). Hypoactivation across the ventrolateral PFC (vlPFC) and orbitofrontal cortex (OFC) have also been reported across different types of anxiety disorders (Etkin et al., 2007; Milad and Rauch, 2007; Killgore et al., 2013). Although these studies demonstrate significant association between prefrontal neural activities and pathological anxiety, in order to understand the etiology of these disorders, it is essential to establish the causal role of the prefrontal cortex in emotion regulation.

Considerable insight into the differential role of subdivisions of medial PFC in the regulation of fear has been gained from studies of fear conditioning and extinction in rodents. In particular, infralimbic mPFC is critical for the extinction of conditioned fear (Morgan et al., 1993; Morgan and LeDoux, 1995; Quirk et al., 2006; Sotres-Bayon et al., 2006) whilst prelimbic mPFC is implicated in the expression of conditioned (Corcoran and Quirk, 2007) and innate fear (Lisboa et al., 2010). A similar dissociation has also been reported in functional neuroimaging studies in humans (Kalisch et al., 2006; Milad et al., 2007a,b). However, much less is known about the role of the ventral regions of PFC in emotion regulation, including OFC and vlPFC.

Experimental studies in monkeys and rodents have provided contradictory reports, with lesions of OFC suppressing (Izquierdo et al., 2005; Kalin et al., 2007; Rudebeck et al., 2007; Machado and Bachevalier, 2008; Fox et al., 2010), enhancing (Izquierdo et al., 2005; Zelinski et al., 2010) or having no effect (Machado et al., 2009; Rudebeck et al., 2013) on negative emotional responses. These discrepancies may be due to differences between studies in the emotional context investigated, i.e., innate fear (Izquierdo et al., 2005; Rudebeck et al., 2006, 2013; Kalin et al., 2007; Machado et al., 2009), conditioned fear (Zelinski et al., 2010), anxiety to a social stimulus (Izquierdo et al., 2005; Rudebeck et al., 2006; Kalin et al., 2007; Machado and Bachevalier, 2008; Fox et al., 2010), or in the type of behavioral response measured, i.e., freezing (Kalin et al., 2007; Fox et al., 2010; Zelinski et al., 2010), complex patterns of anxiety, avoidance and aggression (Izquierdo et al., 2005; Machado and Bachevalier, 2008; Machado et al., 2009), and reward retrieval latency (Izquierdo et al., 2005; Rudebeck et al., 2006, 2013; Kalin et al., 2007; Machado et al., 2009). Alternatively, differences between the specific regions of OFC targeted within monkeys and rodents (Kalin et al., 2007; Zelinski et al., 2010; Rudebeck et al., 2013), or in the method of lesioning, i.e., primarily ablations in monkeys (Izquierdo et al., 2005; Rudebeck et al., 2006; Kalin et al., 2007; Fox et al., 2010) but see (Machado et al., 2009; Rudebeck et al., 2013), and excitotoxic lesions in rodents (Zelinski et al., 2010) may account for the discrepancies. Even less is known of the role of vlPFC in emotion regulation because it has not been studied independently of OFC in monkeys, and whether a homologous area exists in rodents is unclear.

These issues were recently addressed by comparing the effects of excitotoxic lesions, targeting the antOFC (primarily area 11) and vlPFC (area 12) in a new world monkey, the common marmoset. Two distinct tests of negative emotion were studied, Pavlovian discriminative fear conditioning and a test of anxiety typically used in monkeys, the human intruder test. Lesions of both regions resulted in stronger, less adaptable conditioned fear responses and heightened anxiety (Agustín-Pavón et al., 2012), suggesting that both regions contributed independently to the regulation of negative emotion. However, in both tests, the emotional responses were dependent upon learning, since even in the human intruder test the animal's responses are dependent in part, upon their past experiences with humans. This still leaves open the question as to whether a similar heightening of emotional responses would be seen with respect to innate fear.

Innate fear responses are relatively hard-wired and species-specific, and are thought to be of particular relevance to understanding the development of animal phobias in humans (Rosen, 2004). An example is the innate fear response to snakes, fake or real, shown by monkeys bred in captivity and having never been exposed to a snake before (Öhman and Mineka, 2001; Barros et al., 2002; Mineka and Öhman, 2002; Kalin et al., 2007; Shiba et al., 2014). Innate fear shares overlapping but somewhat distinct neural circuitry to that of conditioned fear (Rosen, 2004). Thus, in the present study we determined whether antOFC and vlPFC would also contribute to the regulation of innate fear. We first characterized the behavior and vocalizations of a large cohort of marmosets to a model snake placed into the home cage. Principal Component Analysis (PCA) was used to determine the underlying psychological dimensions (Experiment 1). Next, we investigated the specific effects of either excitotoxic lesions of the antOFC or vlPFC lesion on the animal's response (Experiment 2).

## 2. Materials and methods

All procedures were approved by an Ethical Review Committee from the University of Cambridge and conducted in accordance with the project and personal licenses held by the authors under the United Kingdom 1986 Animals (Scientific Procedures) Act.

### 2.1. Experiment 1: behavioral characterization of responses to a model snake

#### 2.1.1. Subjects

49 naïve common marmosets (*Callithrix jacchus*; 26 females, 23 males, average age 2.7 years ranging 1.8–4.2) were presented with a model snake in their home cage. The data from a subset of these animals (31) had been used in Shiba et al. (2014). All animals were mature young adults in terms of both reproduction (Tardif and Smucny, 2003) and brain morphology (Oga et al., 2013). The animals were housed in male/female pairs in rooms with controlled humidity and temperature and with a 12-h light/dark cycle. They were fed wholemeal bread, hard-boiled egg, and a piece of fruit after testing on weekdays. This diet was supplemented with additional fruit and nuts on the weekends. Water was available *ad libitum*. Prior to receiving the snake test, all animals had been tested on a human intruder test (HIT) [mean interval between the HIT and the snake test: 18.3 ± 14.2 weeks, minimum interval: 2 weeks].

#### 2.1.2. Stimulus

A model snake made of rubber was used as a stimulus. It resembled a cobra and was coiled with its head raised (27 cm in height) and dark brownish in color with black stripes. The model snake was contained in a triangular prism box made of opaque white Perspex (26 cm × 26 cm × 29.5 cm triangle sides × 30 cm high). By removing the sliding door of the box, the snake could be revealed to the subject. The box was designed to conceal the snake from all marmosets except the target subject. The animals had never seen the snake or the box before the experiment.

#### 2.1.3. Test procedures

**Figure 1:**
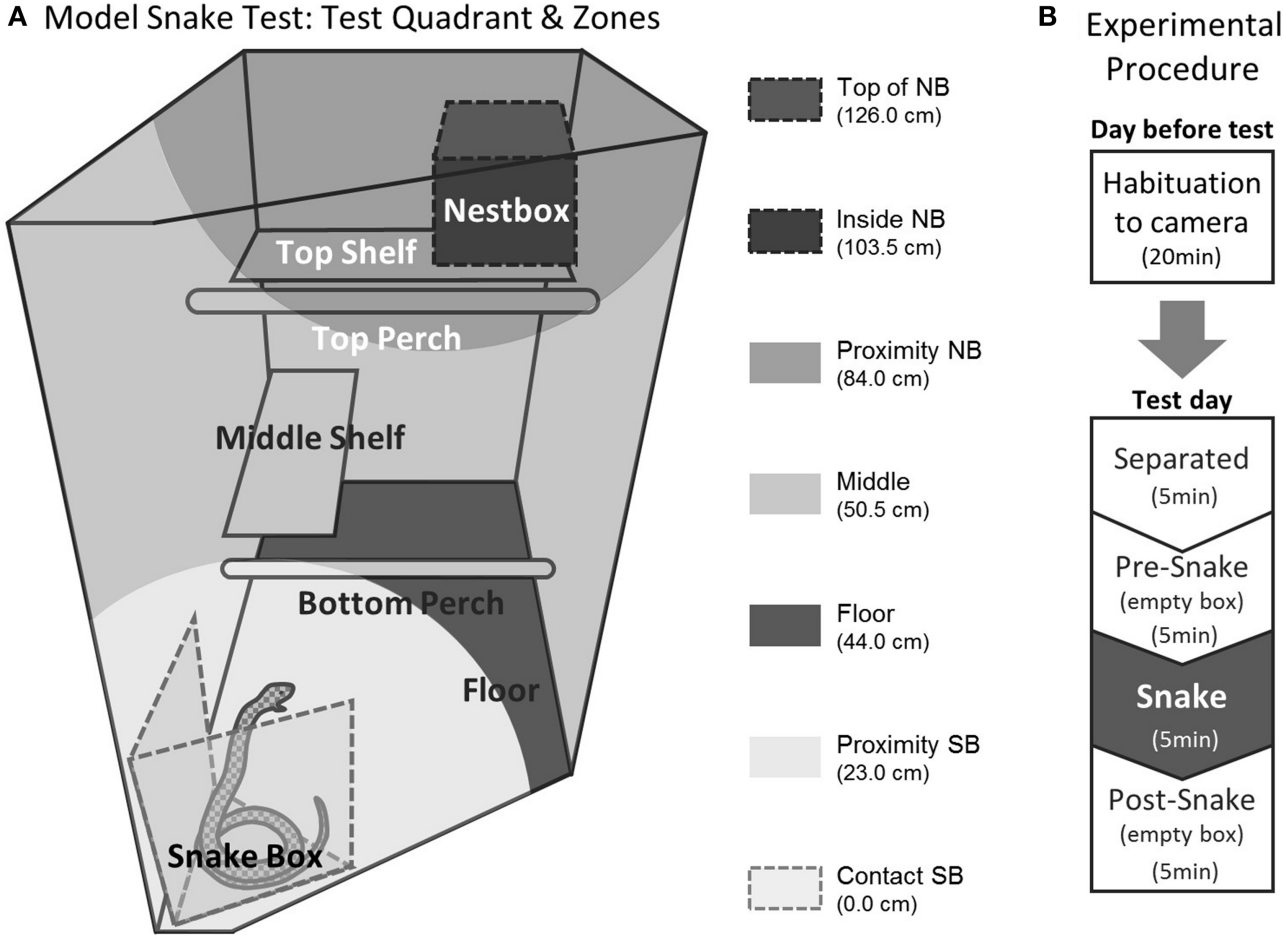
**Experimental setting of the model snake test. (A)** Test quadrant in the home cage. Different shades of gray indicate the seven zones used to calculate the average distance measure. NB; next box, SB; snake box. **(B)** Test procedure. The figure is adapted from Shiba et al. (2014).

Test procedures were identical to the ones previously described (Shiba et al., 2014) but for the purpose of the article, it is fully described here. Habituation and testing took place in the home cage. In both sessions the subject was first separated from the cage mate and restricted to the upper right quadrant (92 cm high × 60 cm wide × 98 cm deep, Figure 1A), preventing visual contact with the cage mate, who was confined to the lower left quadrant. To avoid any aversive contact with the experimenter, the subject was encouraged to enter the quadrant voluntarily. A video camera (Genie CCTV, C5351/12) mounted on a tripod and a shotgun microphone (Pulse, NPM702) were positioned in front of the cage (120 cm and 15 cm from the front, respectively). A second camera (Swann, PPW-245) was positioned above the test quadrant to provide a top-down view. The cameras and microphone were connected to a digital recorder (Pinnacle, Video Transfer) placed outside the room, enabling the experimenter to record the subject's behavior remotely. The 20-min test session was divided into four 5-min phases: “Separated” (only camera and microphone were present), “Pre-snake” (an empty box was placed in the test quadrant), “Snake” (the empty box was replaced with a box containing the model snake) and “Post-snake” (an empty box) (Figure 1B). The habituation session the day before was identical except that the box did not contain the model snake. Testing took place between 12:00–13:00 on weekdays. No more than one animal was tested in the same room on the same day. The order of testing was randomized across the animals.

#### 2.1.4 Behavioral measures

The behavior of each animal was video-recorded and scored by a person blind to the experimental conditions using a quantitative analysis program (JWatcher, Ver. 1.01). For recording the vocalizations, the shotgun microphone was used to ensure that the target animal's calls could easily be distinguished from any other animals' calls in the room. The calls were analyzed with sound spectrogram (Syrinx-PC software, Ver. 2.61). Since many of the behaviors were only displayed in the presence of the snake, only distance and locomotion could be scored across all phases. Inter-rater reliability was assessed by comparing the observers' scores on 15 randomly chosen animals (Table 1). Details of Behavioral parameters are described in Table 1.

**Table 1:** **Behavioral parameters scored during the snake phase**.

**Behavioral Parameter**	**Description**	**Inter-rater reliability**
Average distance from the snake	The test quadrant was divided into seven zones based on the proximity to the snake (Top of nestbox, Inside nestbox, Proximity nestbox, Middle, Floor, Proximity snake box, Contact snake box, Figure 1A). The proportion of time an animal spent in each zone over the 5-min phase was scored. The average distance was obtained by multiplying these proportions with the mean distance of each zone from the snake and summing the products. The distance to the predator stimulus has been shown to be sensitive to anxiolytic treatment (Barros et al., 2000, 2001)	0.99
Locomotion	The proportion of time an animal spent in translational movement over the 5-min phase. The translational movement was registered when an animal altered its body position using all four limbs.	0.84
Stare duration	The proportion of time an animal spent staring at the model snake. Staring was defined as any time when an animal's eyes and head were oriented directly toward the model snake regardless of duration length.	0.79
Stare frequency	The number of discrete occasions on which an animal oriented their eyes and head toward the snake. This measure has been shown to increase in the presence of a predator stimulus compared to a neutral stimulus (Cagni et al., 2011). This measure was included, in addition to stare duration, because some animals spent less time staring at the snake but nevertheless made a high number of short duration “looks” toward the snake.	0.98
Head-cock	Number of head movements rotating the cranium about the rostro-caudal axis of the head itself while the animal's attention is directed toward the snake (Kaplan and Rogers, 2006). This behavior has been reported as an observational behavior (Barros et al., 2002).	0.94
Tsik call	This vocalization has been reported to be an alarm/mobbing call against potential predators (Cross and Rogers, 2006; Bezerra and Souto, 2008; Clara et al., 2008; Cagni et al., 2011) (Supplementary Material Audio 1 Tsik call.wav).	0.99
Tsik-egg call	A tsik call closely followed by an egg call (a short call with a few harmonics). Egg component of this call is associated with vigilance behavior (Bezerra and Souto, 2008) (Supplementary Material Audio 2 Tsik-egg call. wav).	0.99

Other behavioral responses and vocalizations that had previously been reported such as slit stare, scent marking, wet-dog shake, head-body bobbing (rapid movement of the head and body from side to side whilst staring at the object of interest), scratching, barking, phee call, egg call etc. (Stevenson and Poole, 1976; Barros et al., 2000, 2004; Bezerra and Souto, 2008; Agustín-Pavón et al., 2012) were noted. However, these responses were observed so rarely in the presence of the snake stimulus that they were not included in the subsequent analyses.

*Inter-rater reliability was calculated using Pearson correlation coefficients [all p < 0.01 (two-tailed)]*.

#### 2.1.5. Statistical analysis

All analyses were performed using a statistical software SPSS (ver. 17–21). For the “Snake” phase, principal component analysis (PCA) was performed to reduce the separate but correlated measures into weighted composites that reflect underlying psychological dimensions (Field, 2009). Adequacy of sample size for PCA was assessed by the Kaiser-Meyer-Olkin test, which returned an acceptable value of 0.57 (Field, 2009). For PCA, too small correlations between variables are problematic. Bartlett's test for assessing these correlations returned high significance (*p* < 0.0001) ensuring that the correlations between variables are overall significantly different from zero. The component axes are rotated to maximize the loadings of variables onto each component. The paradigm was designed to test the psychological constructs underlying the various behaviors expressed by animals in response to the model snake, which are not completely independent from each other (Field, 2009). Thus, oblique rotation (direct oblimin), that allows correlation between variables, was used to calculate the loadings of the variables on each principal component. Component scores for individual animals were calculated using Anderson-Rubin method (Field, 2009) and used for subsequent descriptive statistics. Pearson's *r* was used to correlate the variables. For the comparison of the average distance and locomotion across the phases, due to the violation of the normality assumption tested by Kolmogorov-Suminov test (distance: “separated,” “snake”; locomotion: “separated,” “pre-snake”), Friedman test was used to compare the means across the phases, and *post-hoc* comparison was performed with Wilcoxon signed-rank test. For correlational analyses, Pearson's *r* was used for the variables that were normally distributed or Spearman's ρ was used for those that violated the normality assumption.

### 2.2. Experiment 2: effect of antOFC and vlPFC excitotoxic lesions on the behavioral responses to the model snake

#### 2.2.1. Subjects

14 marmosets (5 females, 9 males, average age 3.2 years ranging 2.0–4.1) that were not included in Experiment 1, were used. The housing condition and diet were the same as described in Section 2.1.1. All of the animals had experience of a discriminative fear conditioning paradigm and HIT as part of a previously reported behavioral study (Agustín-Pavón et al., 2012) [mean interval between the fear conditioning and the snake test: 40.9 ± 24.8 weeks, minimum interval: 14.9 weeks; mean interval between the HIT and the snake test: 13.2 ± 4.2 weeks, minimum interval 3.1 weeks]. Four of them (1 female, 3 males) had received excitotoxic lesions of antOFC and five of them (2 females, 3 males) had received excitotoxic lesions of vlPFC. The remaining five (2 females, 3 males) were sham-operated controls. The lesions were made following training on a conditioned fear discrimination task. After surgery, animals first received further fear discrimination training and testing, then received the HIT and finally, as reported here, received the model snake test. Mean interval between the surgery and test was 39 ± 7 weeks, equally varied across groups [Levene's test of homogeneity of variance: *F*_(2, 11)_ = 2.46 *p* = 0.131].

#### 2.2.2. Surgery

**Figure 2:**
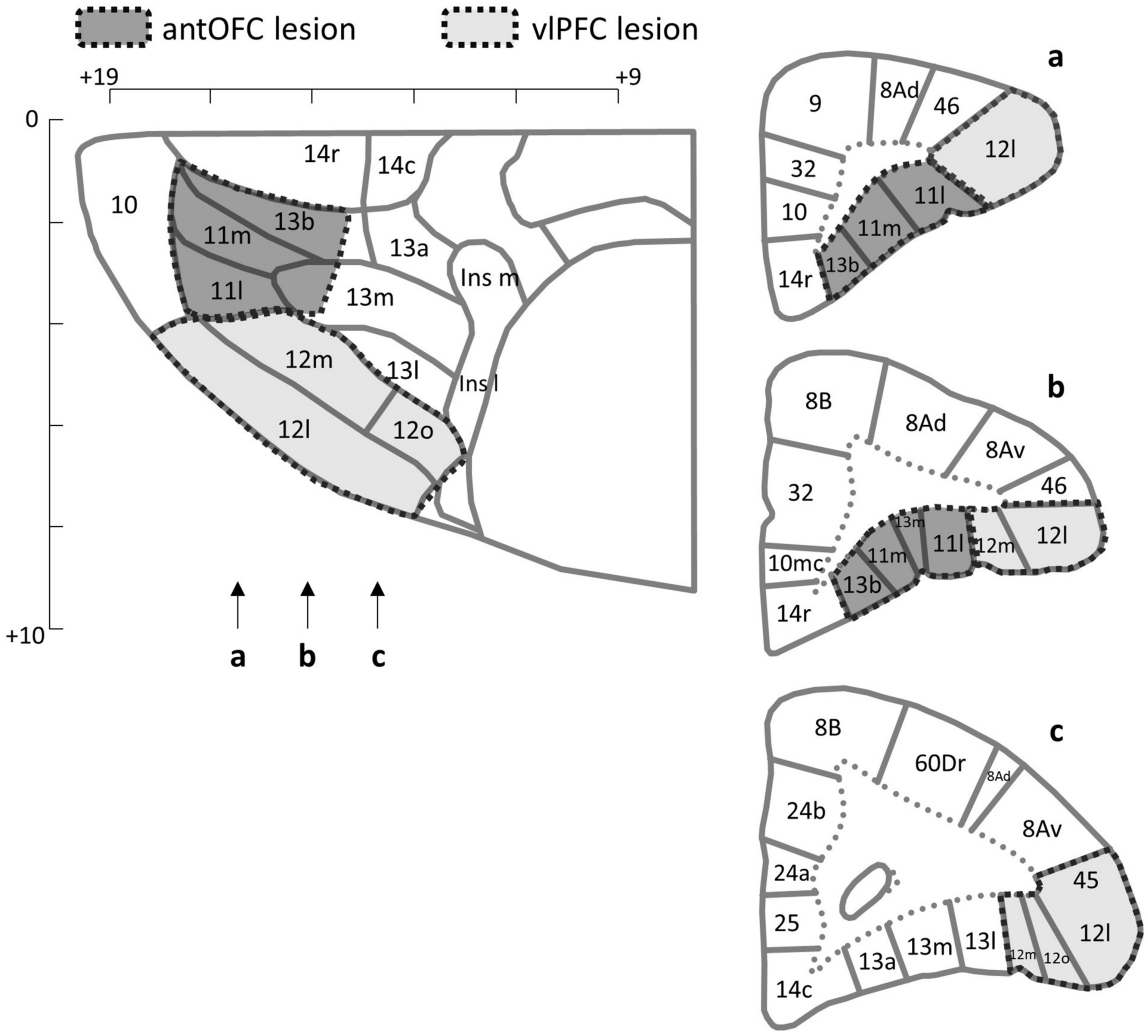
**Lesion targets**. The horizontal (top left) and coronal (right) planes of the frontal cortex of the marmoset. Arrows indicate the levels of sections in the coronal plane. Areas targeted for the excitotoxic lesion are indicated for the antOFC (dark shade) and vlPFC (light shade). Numerical designations reflect likely homologous regions with the macaque monkey. m, Medial. l, Lateral. Ins, Insula. Scale bar = 1 mm. The templates are adapted from Burman and Rosa (2009).

Surgical procedures have been described in an earlier report (Agustín-Pavón et al., 2012). All surgical procedures were performed under aseptic conditions. The animals were premedicated with ketamine hydrochloride (sedative, 0.1 ml of a 100 mg/ml solution, intramuscular (i.m.); Amersham Pharmacia and Upjohn, Piscataway, NJ) and carprofen (prophylactic analgesic, 0.03 ml, subcutaneous (s.c.)), and anesthetized by isoflurane intubation (flow rate 2–2.5%; IsoFlo, Abbott Laboratories, Abbott Park, IL). The animals were placed into a stereotaxic frame (David Kopf, Tujunga, CA) with their head securely fixed in position with specially modified incisor and zygoma bars. A standardization technique (Roberts et al., 2007) was used to determine the appropriate injection sites for each animal independently, based on the thickness of the marmoset's frontal pole. Excitotoxic lesions of the antOFC and vlPFC were then made by infusing 0.4–1.6 μl/site of a 0.09 M solution of quinolinic acid bilaterally into six/seven sites (Figure 2). For all placements, infusions were made at a rate of 0.1 μl/20 s by using a 2- μl precision Hamilton sampling syringe (Precision Sampling, Baton Rouge, LA) through a stainless-steel cannula (30 gage). The cannula remained in place for 4 min, after which it was withdrawn by 1 mm, where it remained for an additional 2 min before being slowly removed from the brain. The skin was sutured and covered with a protective barrier (Germoline New Skin; Bayer, Newbury, UK), and dexamethasone phosphate (0.2 ml i.m.; Fauling Pharmaceuticals plc, Warwicks, UK) was given to avoid the unlikely event of tissue inflammation. The animals received diazepam Syrup (3–10 mg/kg oral, Sando, Princeton Township, NJ) as required within the first 24 h to suppress epileptic seizure activity; although this was rare. Non-steroidal analgesics (0.1 ml Metacam oral; St. Joseph, MO) were given for 3 days after surgery at 24-h intervals. Sham-operated control animals underwent the same surgical procedure as lesioned animals, except that they received infusions of sterile phosphate buffer vehicle, into the antOFC (*n* = 2) or vlPFC (*n* = 3). The animals had at least a 2-week recovery period before behavioral testing.

#### 2.2.3. The model snake test

The animals were tested on the model snake test as described in Section 2.1.3. The behavioral responses displayed to the model snake were scored and analyzed as described in Section 2.1.4.

#### 2.2.4. Statistical analysis

SPSS (ver. 17–21) was used to carry out statistical analyses. To calculate the component scores of each animal, first, behavioral scores were standardized using the mean and standard deviation of all experimental groups, then, the component score coefficients obtained from the PCA with the larger sample (*n* = 49, Section 2.1.5) were applied to the z-scores and the products were summed for each component (Agustín-Pavón et al., 2012). Two-Way factorial ANOVA was used to compare the derived component scores between the groups. For the raw score of each behavioral measure, Kolmogorov-Sminov test was used to test the normality assumption, and Levene's test was used to examine the homogeneity of variance. One-Way ANOVA was used to compare each behavioral measure between the groups. For those violating the normality assumption (tsik call), the non-parametric Kruskal-Wallis test and Mann-Whitney test were used to compare the scores between the groups. Mixed design ANOVA was used to compare the average distance and locomotion scores of the groups across the four phases.

#### 2.2.5. Histological analysis

The histological procedures were described in an earlier report (Agustín-Pavón et al., 2012). All animals were euthanized with Dolethal (1 ml of a 200 mg/ml solution, pentobarbital sodium, i.p.; Merial Animal Health, Essex, U.K.). Animals were then perfused transcardially with 500 ml of 0.1 M PBS (pH 7.4), followed by 500 ml of 0.4% formaldehyde-buffered solution, washed through over 10 min. The entire brain was removed and placed in fixative solution overnight before being transferred to a 30% sucrose solution in 0.01 M PBS for a minimum of 48 h. For verification of lesions, coronal sections (60 μm) of the brain were cut by using a freezing microtome and stained with cresyl fast violet. The sections were viewed under a Leitz DMRD microscope (Leica Microsystems, Wetzlar, Germany), and lesioned areas were defined by the presence of major neuronal loss, often with marked gliosis.

## 3. Results

### 3.1. Experiment 1: behavioral characterization of the model snake test

**Figure 3:**
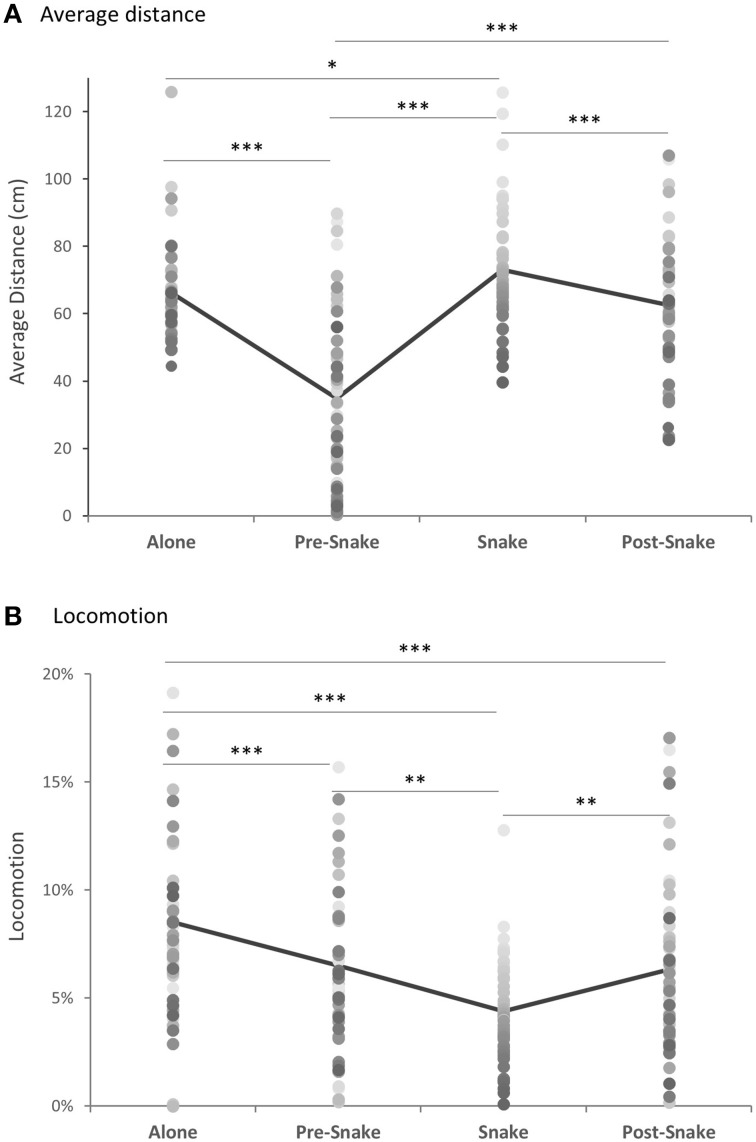
**(A)** Average distance from the position of the snake box and **(B)** locomotion, across all phases of the model snake test (*n* = 49). Gray-shaded dots represent individual animals. ^*^p < 0.05, ^**^p < 0.01, ^***^p < 0.001.

There were significant differences in average distance and locomotion across the four phases. As expected, animals maintained a greater distance from the front corner of the cage where the white box was positioned when it contained the rubber snake (Figure 3A). They also showed reduced locomotion during that phase (Figure 3B). In contrast, in the pre-snake phase the majority of animals moved close to the white box and in many cases, climbed on top of it and explored inside. In the post-snake phase greater distance was maintained from the box than in the pre-snake phase, presumably as a consequence of experience with the snake, but was, nevertheless, reduced compared to the snake phase. There was marked individual variation, both in response to the initial introduction of the white box and subsequently to the presence of the snake. There was a weak but significant tendency for animals that maintained the greatest distance from the snake to be the same animals that maintained the greatest distance from the white box in the pre-snake phase. This suggests that the novel white box may also have induced a mild state of anxiety.

When distance and locomotion were compared across the four phases, there was a main effect of phase for both measures [Friedman Test: for distance, χ^2^_(3)_ = 71.25, *p* < 0.0001, for locomotion, χ^2^_(3)_ = 33.67, *p* < 0.0001] (Figures 3A,B). *Post-hoc* analysis revealed that the average distance from the white box was greatest when it contained the snake and significantly different from all other phases [Wilcoxon singed-rank test, “snake” vs. “separated” *Z* = −2.33, *p* = 0.02, “snake' vs. “pre-snake” *Z* = −5.95, *p* < 0.0001, “snake” vs. “post-snake” *Z* = −3.48, *p* < 0.001]. In contrast, the majority of animals approached and touched the empty white box during the pre-snake phase [“pre-snake” vs. “alone” *Z* = −5.58, *p* < 0.0001], but less so following snake exposure [“pre-snake” vs. “post-snake” *Z* = −3.48, *p* < 0.0001]. There was also a significant positive correlation between the pre-snake and snake phases [Spearman's ρ = 0.42, *p* = 0.003].

For the locomotion, the animals were least mobile in the presence of the snake [“snake” vs. “alone” *Z* = −4.98, *p* < 0.0001, “snake” vs. “pre-snake” *Z* = −2.75, *p* = 0.006, “snake” vs. “post-snake” *Z* = −3.19, *p* = 0.001] and most locomotive during the separated phase [“separated” vs. “pre-snake” *Z* = −3.44, *p* = 0.001, “separated” vs. “post-snake” *Z* = −3.51, *p* < 0.001]. The locomotion between the “pre-snake” and “post-snake” did not differ significantly [“pre-snake” vs. “post-snake” *Z* = −0.40, *p* = 0.69].

**Figure 4:**
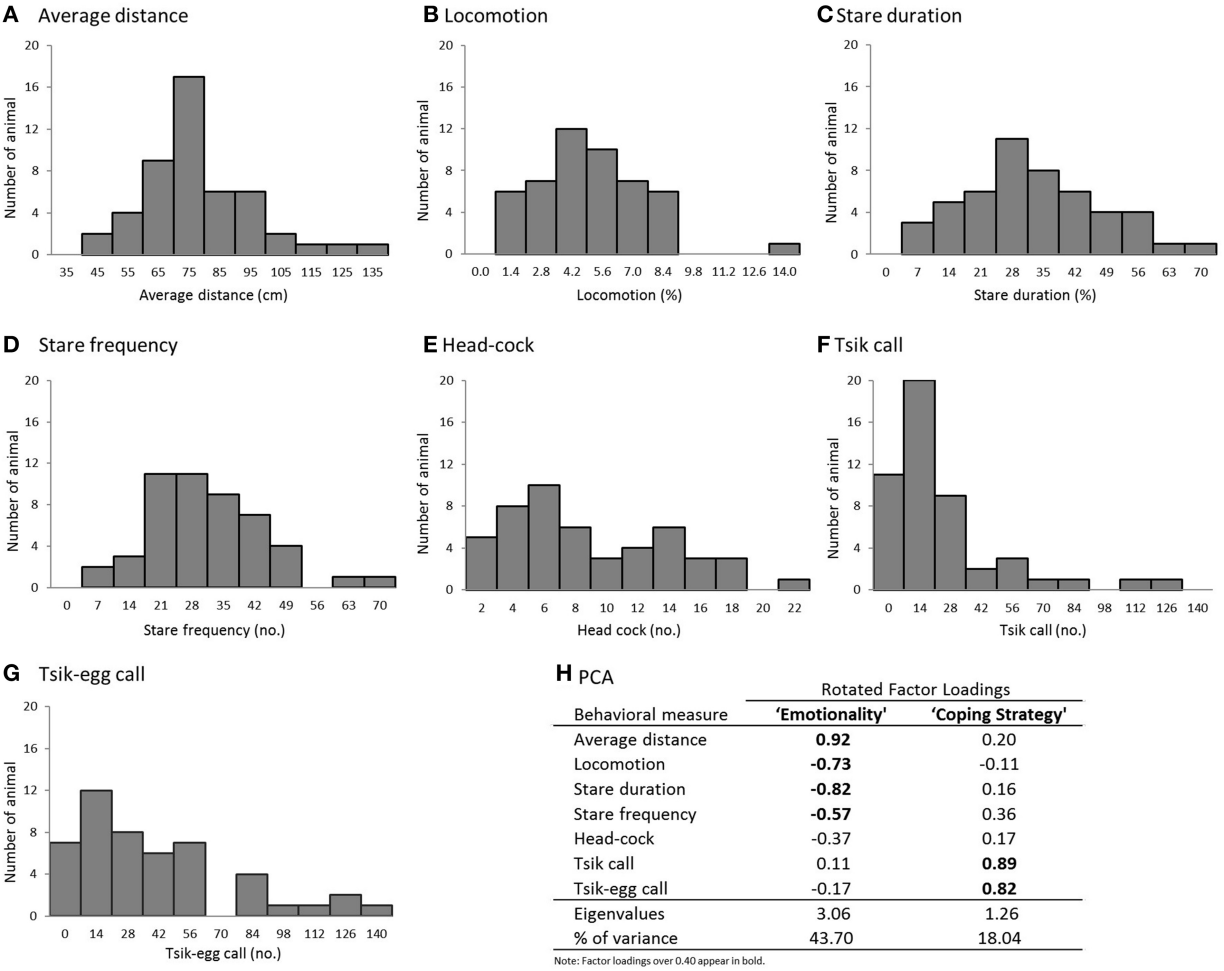
**(A–G)** Histograms of the behavioral measures in the model snake test (*n* = 49). **(A)** average distance from the snake, **(B)** time spent in translational locomotion, **(C)** duration of staring at the snake, **(D)** number of discrete “looks” toward the snake, **(E)** number of head-cocks, **(F)** number of tsik calls, and **(G)** number of tisk-egg calls. **(H)** PCA results (Pattern Matrix).

In the presence of the snake there were an additional repertoire of behaviors observed, including head cocks and vocalizations, that were not observed in other phases. These are depicted individually in Figure 4 and described in detail in Table 2.

**Table 2:** **Behavioral responses during the snake phase**.

**Behavioral parameter**	**Mean**	**Standard deviation**	**Description**
Average distance from the snake (cm) (Figure 4A)	73.05	18.34	Most animals stayed away from the snake, positioning themselves either in the middle of the cage or further back, close to the nestbox. No animal touched the snake and only a few ventured into the zone proximal to the snake. A small number of animals (*n* = 3) stayed on top of the nestbox for the majority of the time, the furthest point from the snake.
Locomotion (%) (Figure 4B)	4.38	2.37	In the presence of the snake, most animals spent a relatively small proportion of time in translational movement although no animal was completely immobile during the entire 5-m period. It is worth noting that the animals that showed the greatest reduction of locomotive activity (7 animals) also made no vocalizations.
Stare duration (%) (Figure 4C)	29.24	14.73	Many animals spent nearly a third of the 5-m period staring at the snake with 20% spending more than half their time staring at the snake.
Stare frequency (events) (Figure 4D)	28.78	12.84	A significant positive correlation between stare duration and frequency [Pearson's *r* = 0.67, *p* < 0.001] indicate that those that made fewer short duration “looks” at the snake may have been avoiding eye-contact with it.
Head-cock (events) (Figure 4E)	8.39	5.13	This measure was highly variable across individuals as can be seen by the non-normal distribution of the histogram.
Tsik call (events) (Figure 4F)	17.88	27.19	Not all animals displayed this vocalization in the presence of the snake (22% made none). Of those that did, 59% made up to 28 tsik calls, whilst a few (8%) produced 70 or more calls.
Tsik-egg call (events) (Figure 4G)	34.24	34.84	The pattern of tsik-egg calls was similar to that of the tsik calls with some animals making none (14%) whilst a few (18%) made a large number (>70). However, the animals that made a large number of tsik-egg calls didn't necessarily make large numbers of tsik calls and vice versa.

To understand the structure of the behavioral repertoire displayed in the presence of the snake and to elucidate possible underlying psychological dimensions, a PCA was conducted on the seven behavioral variables with oblique rotation. An initial analysis was run to obtain eigenvalues for each component in the data. Two components had eigenvalues over Kaiser's criterion of 1 and in combination explained 61.74% of the total variance; therefore, these components were retained for the final analysis. Figure 4H shows the factor loadings after rotation.

The behaviors that loaded highly on component 1 included average distance, locomotion, stare duration and stare frequency. Those marmosets with the highest component 1 score displayed reduced locomotor activity; avoided visual contact with the snake; and maintained a greater distance from the snake, suggesting that this component represents an overall level of emotionality (i.e., anxiety/fear). A similar pattern of variable loadings on the emotionality component was reported in the human intruder paradigm (Agustín-Pavón et al., 2012). The behaviors that loaded on component 2 were primarily vocalizations: tsik and tsik-egg calls, such that marmosets with the highest score made the greatest number of tsik and tisk-egg calls. Tsik calls are primarily mobbing calls, made in the presence of conspecifics from other social groups, predator threat and unfamiliar humans. The calls function to solicit the attention of other marmosets so the group can act together to drive the predator away (Bezerra and Souto, 2008). Tsik calls not only act to reduce cortisol levels of the animal that emits them, but also of other animals around (Clara et al., 2008). Overall, this call is an active coping response made by an animal when it faces a threatening situation. The tsik-egg call has been described together with egg calls, which are associated with vigilance behavior, in potentially threatening contexts (Bezerra and Souto, 2008). Thus, component 2 most likely represents the coping strategy displayed by the marmoset in a threatening situation.

### 3.2. Experiment 2: effect of antOFC and vlPFC lesions on the behavioral responses to the model snake

#### 3.2.1. Histology of excitotoxic antOFC and vlPFC lesions

**Figure 5:**
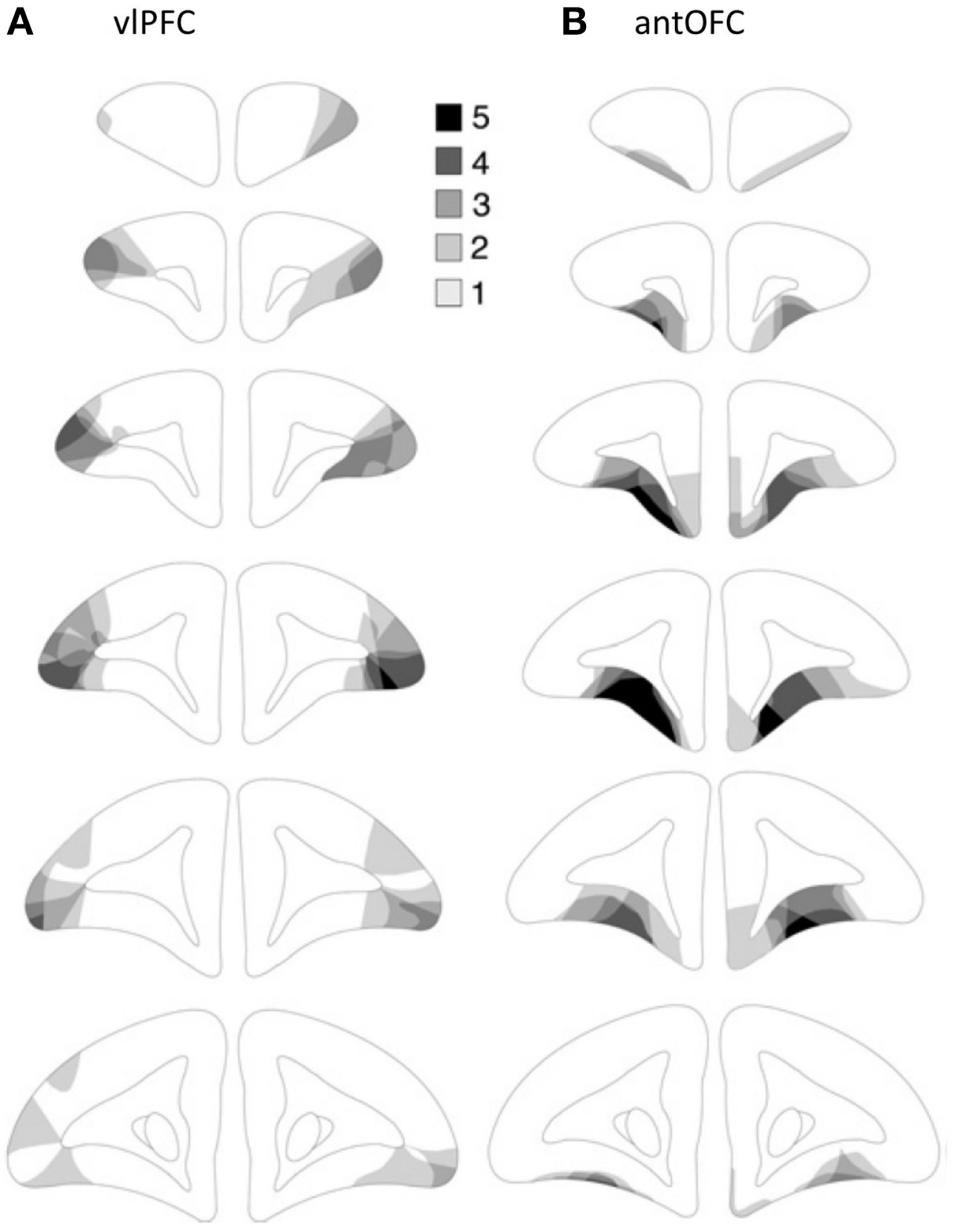
**Excitotoxic lesions of (A) vlPFC and (B) antOFC**. Schematic coronal sections taken through the frontal lobe (anterior-posterior) of the marmoset. The five decreasing shades of gray indicate regions that were lesioned in all five, four, three, two or one animal, respectively. The diagrams are adapted from Agustín-Pavón et al. (2012).

For each animal, areas with cell loss were schematized onto drawings of standard marmoset coronal sections, and composite diagrams were then made to illustrate the extent of overlap between lesions (Figure 5). All animals in the vlPFC lesioned group sustained neuronal cell loss within the vlPFC (Area 12/45) although the cell loss varied in its rostro-caudal extent between animals. Only in one animal was there some encroachment into the antOFC region, unilaterally. In the antOFC lesion group, most animals sustained marked neuronal loss throughout area 11 and the anteromedial portion of area 13. Only in one animal was there significant neuronal loss, unilaterally, in area 14. No obvious behavioral differences between animals within the lesion groups were seen.

#### 3.2.2. Both antOFC and vlPFC lesions resulted in heightened “emotionality” and reduced “coping strategy” scores compared to the control group

**Figure 6:**
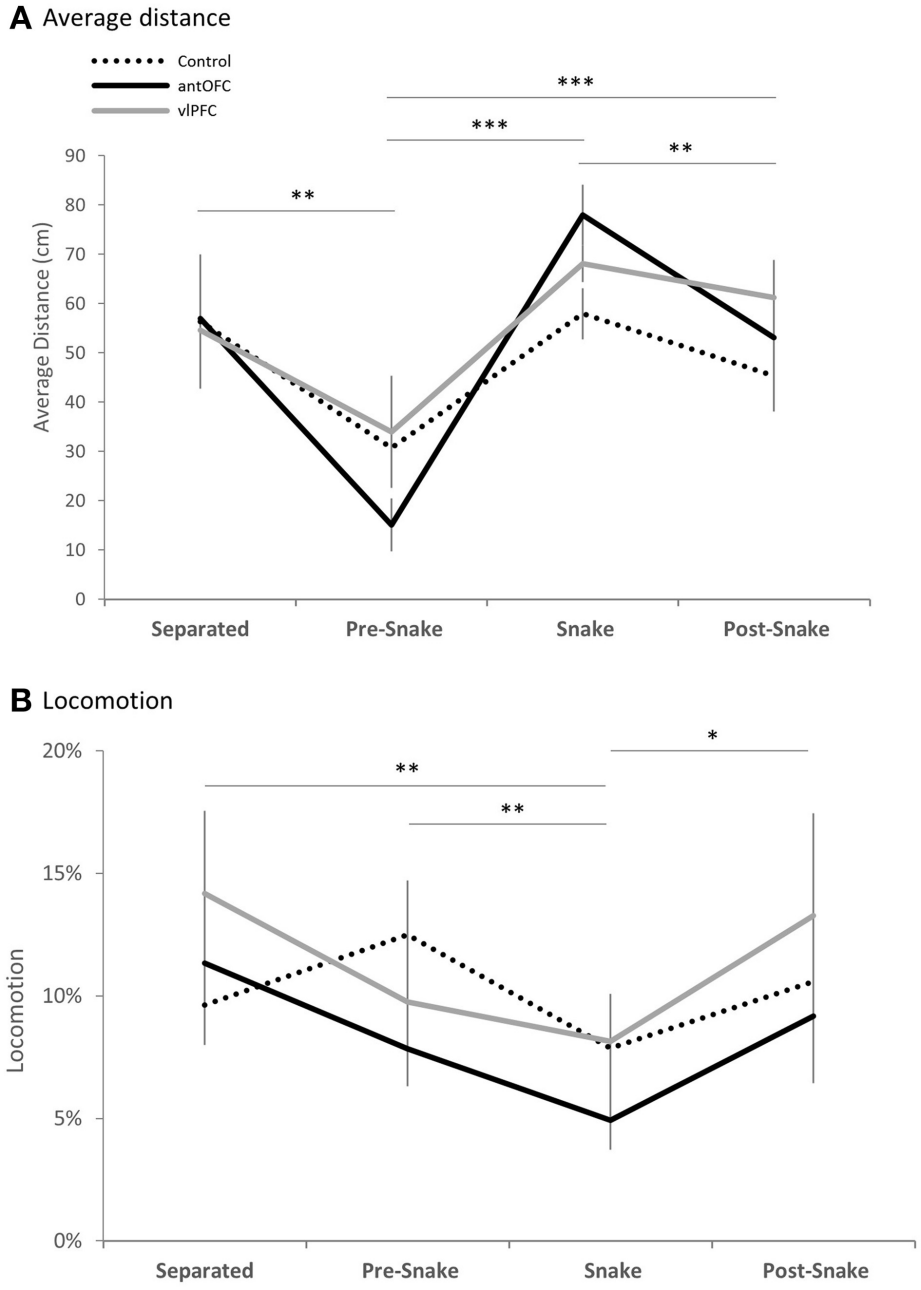
**Phase comparison of (A) average distance from the snake stimulus and (B) locomotion measure of all three groups** (dotted line: control, black line: antOFC, gray line: vlPFC). Error bar shows standard error of the mean (SEM). ^*^p < 0.05, ^**^p < 0.01, ^***^p < 0.001.

All animals exhibited withdrawal responses in the presence of the snake. When compared across the four phases, average distance from the white box was greatest when it contained the snake, across all three groups [mixed-design ANOVA (Phase, Group), main effect of Phase: *F*_(3, 33)_ = 19.46, *p* < 0.001; *post-hoc* pairwise comparison of Phase: “snake” vs. “pre-snake” *p* < 0.001, “snake” vs. “post-snake” *p* = 0.008] (Figure 6A). Locomotion was also significantly reduced in all groups during the snake phase compared to all other phases [mixed-design ANOVA (Phase, Group), main effect of Phase: *F*_(3, 33)_ = 6.10, *p* = 0.002; *post-hoc* pairwise comparison of Phase: “snake” vs. “separated” *p* = 0.003, “snake” vs. “pre-snake” *p* < 0.001, “snake” vs. “post-snake” *p* = 0.012] (Figure 6B).

**Figure 7:**
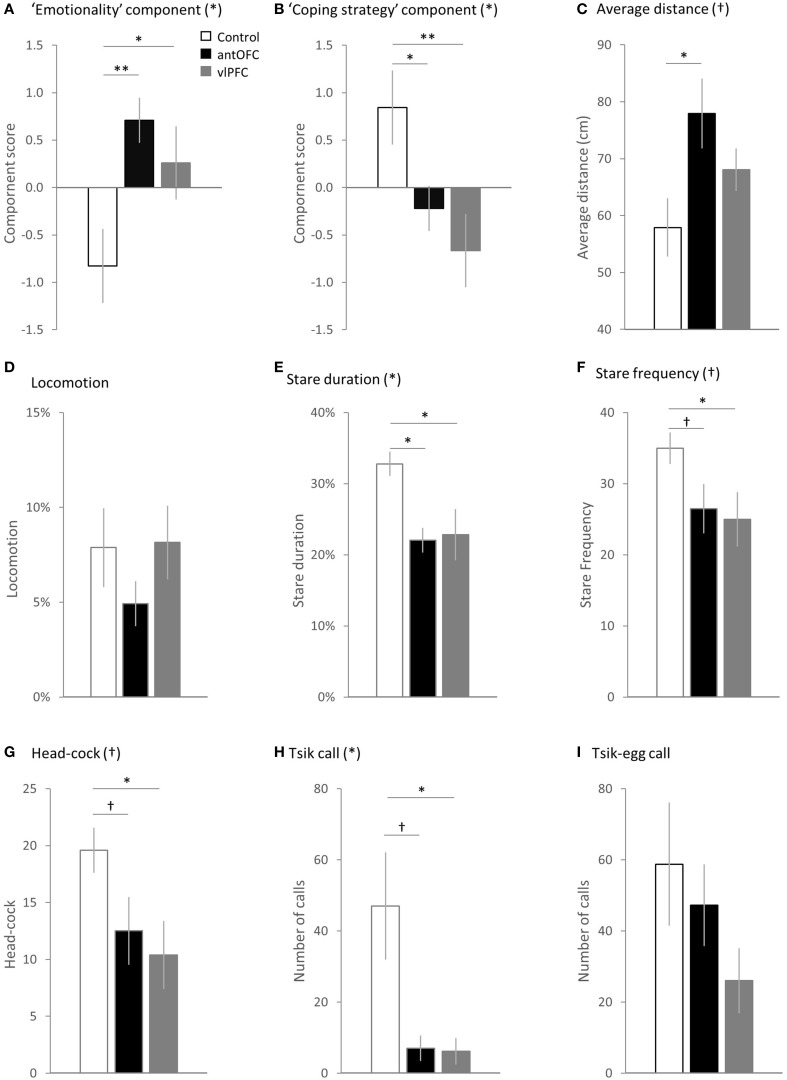
**Comparison between the groups (white bar: control, black bar: antOFC, gray bar: vlPFC) in the principal components and individual variables. (A)** “Emotionality” component, **(B)** “coping strategy” component, **(C)** average distance from the snake stimulus, **(D)** time spent in translational locomotive movement, **(E)** duration of staring at the snake, **(F)** number of discrete “looks” toward the snake, **(G)** number of head-cocks, **(H)** number of tsik calls, and **(I)** number of tisk-egg calls. Error bar shows SEM. (^*^) and (†) denote a significant and trend level main effect of the group respectively. ^†^*p* < 0.1, ^*^p < 0.05, ^**^p < 0.01.

During the snake phase, both antOFC and vlPFC lesioned groups displayed significantly higher overall “emotionality” component scores in response to the snake than did the controls [Two-Way factorial ANOVA (Group, Component), Group × Component interaction *F*_(2, 11)_ = 12.65, *p* = 0.001, *post-hoc* pairwise comparison for “emotionality” component: “antOFC” vs. “control” *p* = 0.007, “vlPFC” vs. “control” *p* = 0.030] (Figure 7A). There was no significant difference between the lesioned groups [post-hoc pairwise comparison for “antOFC” vs. “vlPFC” *p* = 0.354]. In particular, the antOFC lesioned group displayed a strong trend for increased distance from the snake [One-Way ANOVA, *F*_(2, 11)_ = 3.90, *p* = 0.052; *post-hoc* pairwise comparison for “control” vs. “antOFC” *p* = 0.018] (Figure 7C), both antOFC and vlPFC groups avoided staring at the snake [One-Way ANOVA, *F*_(2, 11)_ = 5.35, *p* = 0.024; *post-hoc* pairwise comparison for “control” vs. “antOFC” *p* = 0.016, “control” vs. “vlPFC” *p* = 0.018] (Figure 7E) and the vlPFC lesioned group tended to display fewer investigative “looks” at the snake [One-Way ANOVA, *F*_(2, 11)_ = 2.92, *p* = 0.096; *post-hoc* pairwise comparison for “control” vs. “vlPFC” *p* = 0.045] (Figure 7F) and fewer head-cocks [One-Way ANOVA, *F*_(2, 11)_ = 3.45. *p* = 0.069; *post-hoc* pairwise comparison for “control” vs. “vlPFC” *p* = 0.028] (Figure 7G). The groups did not significantly differ in locomotion [One-Way ANOVA, *F*_(2, 11)_ < 1] (Figure 7D).

In addition, both antOFC and vlPFC lesioned groups displayed a significantly reduced “coping strategy” component score compared to the control group [Two-Way factorial ANOVA (Group, Component), Group × Component interaction *F*_(2, 11)_ = 12.65, *p* = 0.001, *post-hoc* pairwise comparison for “coping strategy” component: “antOFC” vs. “control” *p* = 0.039, “vlPFC” vs. “control” *p* = 0.005] (Figure 7B). There was no significant difference between the lesioned groups [*post-hoc* pairwise comparison for “antOFC” vs. “vlPFC” *p* = 0.351]. Notably, both lesioned groups emitted a significantly fewer number of proactive tsik calls than did controls [non-parametric Kruskal-Wallis Test, *H*_(2)_ = 6.19, *p* = 0.045; *post-hoc* pairwise comparison Mann-Whitney Test, “antOFC” vs. “control” *U* = 2.00, *p* = 0.050, “vlPFC” vs. “control” *U* = 2.00, *p* = 0.027] (Figure 7H). The groups did not significantly differ in the number of tsik-egg calls [One-Way ANOVA, *F*_(2, 11)_ = 1.65, *p* = 0.237] (Figure 7I).

## 4. Discussion

Marmosets showed a relatively consistent pattern of behaviors in response to the presence of a predator threat, i.e., model snake, in their home cage (Experiment 1), although the extent to which individual animals displayed these behaviors differed quite considerably. Seven distinct behaviors and vocalizations were identified. PCA revealed two underlying components, which were labeled “emotionality” and “coping strategy,” based on the pattern of behaviors and vocalizations loading on each of the components. Compared to the sham-operated controls, marmosets with excitotoxic lesions of either vlPFC or antOFC had significantly higher “emotionality” scores, reflecting the animal's heightened anxiety/fear-related responses to the snake (Experiment 2). The lesioned animals also had a reduced “coping strategy” score. In particular, they emitted markedly fewer mobbing calls than controls. These results support the hypothesis that ventral PFC plays a role, not only in regulating learned fear and anxiety, as shown in our previous study (Agustín-Pavón et al., 2012), but also in regulating innate fear to predator threat.

Fear of snakes has been widely exploited to induce anxiety/fear responses experimentally in primates. Compared to the periods before and after exposure to the snake, during the snake presentation, all marmosets displayed an avoidance response, spending more time at the back half of the cage and showing reduced locomotion. They also displayed varied levels of “attentional” responses directed at the snake, in the form of head cocks (Menzel, 1980; Kaplan and Rogers, 2006) and stares. Particularly varied of the responses however, was the number of mobbing calls that an animal produced, indicative of whether they were engaging in an active or passive coping strategy (Cross and Rogers, 2006; Agustín-Pavón et al., 2012). Marmosets have been observed to produce this mobbing call also in the presence of a human intruder (Agustín-Pavón et al., 2012), however, both the numbers of calls (mean: HIT: 5.08 ± 1.57, Snake: 17.88 ± 3.88) and the numbers of animals producing this call (HIT: 16.3%, Snake: 40.8% of all animals tested) were far greater in response to the snake. Moreover, those animals that made the most mobbing calls in the presence of the snake were not the same animals that made large numbers of mobbing calls in the presence of the human intruder suggesting the relative independence of an animal's coping strategy in the two distinct contexts. This is further supported by marked differences in other responses between the two aversive tests including egg calls, which were often observed with head and body bobbing behavior in the presence of the human intruder (Agustín-Pavón et al., 2012) but not snakes and vice versa for head cocks. Together, these differences highlight the stimulus-specific behavioral responses displayed by marmosets to predator threat i.e., snakes and ambiguous social stimuli, i.e., human intruders.

Marked individual variability between marmoset's behavioral emotionality responses to the snake has been reported previously and proposed to represent a spectrum of anxiety trait present within a population (Shiba et al., 2014). Trait anxiety refers to a general tendency to perceive and react negatively in a wide variety of stressful situations (Gaudry et al., 1975). We have recently shown that high scorers on the emotionality component of the snake test also display high scores on the equivalent component on the HIT (Mikheenko et al., in press), further supporting the proposal that the considerable variation in the observed responses reflects a stable, trait-like anxiety in marmosets.

The finding that selective lesions of the antOFC and vlPFC in marmosets heightened the emotional responses to predator threat is consistent with the heightened emotionality they displayed to a human intruder (Agustín-Pavón et al., 2012). Overall, the lesioned animals spent more time at the back of the cage and less time engaged in locomotion, compared to controls. Their number of head cocks also increased and they spent less time “looking/staring” at the snake. This overall pattern of behavior is very similar to that seen in high trait-anxious marmosets (Mikheenko et al., in press). However, these results differ from those of previous studies investigating the contribution of primate ventral PFC to responsivity to predator threat. Relatively large aspirative lesions that included the ventrolateral area 47/12, as well as orbital areas 11, 13, and 14 led to reduced fear of a real or fake snake; with lesioned animals being quicker to retrieve food reward in the presence of a snake than unoperated controls (Kalin et al., 2007). Such blunting of the fear response and reduction of food retrieval latencies has also been reported after large aspirative lesions of ventral PFC that spared ventromedial area 14 (Rudebeck et al., 2006) and after more restricted aspirative lesions of OFC (areas 11, 13, 14, and 10, sparing 47/12) (Izquierdo et al., 2005). In contrast, aspirative lesions confined to areas 11 and 13 of the OFC (along with anterior agranular insular) (Machado et al., 2009) or excitotoxic lesions of areas 11, 13, and 14 (Rudebeck et al., 2013) left food retrieval latencies in the presence of a snake, intact, i.e., they exhibited increased latencies in the presence of a snake, similar to that seen in controls. The most likely explanation for blunting of the fear responses with large aspirative lesions is that removal of such a large area of ventral PFC is accompanied by damage to fibers of passage on their way to and from adjacent prefrontal regions, e.g., dorsal and lateral PFC, including monoaminergic afferents. Such gross damage may well lead to an overall reduction in arousal and corresponding blunting of affective responses. That such effects are attributable to damage of fibers of passage is supported by the recent finding that ablation of a small strip of tissue in the posterior OFC (that was included in the original large aspirative lesions, Izquierdo et al., 2005; Rudebeck et al., 2006) also leads to blunting of the fear response (Rudebeck et al., 2013). Less easily explained are the complete lack of effects of smaller aspirative or excitotoxic lesions of multiple sectors of the OFC regions. One plausible explanation is that distinct OFC regions have opposing contributions, with lesions of both acting to mask each other' effects. Such an opposing behavioral pattern has been seen when comparing selective (and combined) lesions of medial orbital and lateral orbital regions of the OFC in rats on their ability to select between immediate and delayed reward (Mar et al., 2011). Whether a similar opposing pattern is seen in primate OFC remains to be determined. Nevertheless, results from the present study reveal that the antOFC (area 11, 13b, see Figure 2) is implicated in down-regulatory control of innate fear responses.

Given that the OFC, including areas 11 and 13, send projections to the GABAergic intercalated cells within the amygdala, which in turn issue inhibitory projections to the central nucleus (Ghashghaei and Barbas, 2002), the amygdala is the most likely target of orbitofrontal down-regulatory control. Lesions to the amygdala in monkeys reliably impair the fear response to a snake (Aggleton and Passingham, 1981; Zola-Morgan et al., 1991; Meunier et al., 1999; Kalin et al., 2001, 2004; Prather and Lavenex, 2001; Amaral et al., 2003; Stefanacci et al., 2003; Izquierdo and Murray, 2004; Izquierdo et al., 2005; Mason et al., 2006; Machado et al., 2009), an effect that has been replicated in a human with a focal bilateral lesion of the amygdala (Feinstein et al., 2011). Similarly, human neuroimaging studies of specific phobias, including snake phobia, consistently report hyper-activation of the amygdala to threat relevant stimuli (see reviews: Etkin et al., 2007; Linares and Trzesniak, 2012). Moreover, such an enhanced amygdala response to the feared stimulus is often associated with altered activation in the OFC, (Carlsson et al., 2004; Ohman, 2005; Ahs et al., 2009; Linares and Trzesniak, 2012) supporting the hypothesis of orbitofrontal regulatory control over the amygdala.

Besides the contribution of antOFC to regulation of emotional responses to predator threat our current study also demonstrated that lesions of the vlPFC, independently from that of the antOFC, result in enhanced anxiety/fear-related responses. This is consistent with our previous finding that selective excitotoxic lesions of the vlPFC resulted in less adaptable conditioned fear responses, overall heightened behavioral and autonomic responses in fear discriminative conditioning and enhanced anxiety-related behaviors in response to a human intruder (Agustín-Pavón et al., 2012). The role of vlPFC in the regulation of negative emotion has been less well explored in comparison to the OFC. However, given its reciprocal connectivity with the amygdala, albeit less robust than that of the OFC (Ghashghaei et al., 2007), as well as the input of object-processed visual information (Kringelbach and Rolls, 2004; Barbas, 2007), the vlPFC is in a good position to exert regulatory control in a threat encounter. Certainly, patients with generalized anxiety disorder exhibit increased activation in the vlPFC to an angry facial expression which is negatively correlated with anxiety symptom severity (Monk et al., 2006) suggesting that this activation serves as a compensatory response. Moreover, when healthy humans are presented with highly aversive and arousing pictures and instructed to suppress the induced negative affect by means of reappraisal, this inhibition of negative affect is associated with increased vlPFC activation, which is inversely correlated with amygdala activity (Ochsner et al., 2002; Phan et al., 2005). Finally, unpublished findings from our lab implicate this region in negative decision making in an approach-avoidance task with lesions resulting in an increased avoidance response (Clark et al, SFN abstract 2014). Given that this same region is also implicated in the ability to shift attentional sets both in marmosets (Dias et al., 1996) and humans (Hampshire and Owen, 2006) the observed increase in fear/anxiety responses following lesions to this region may be a consequence of enhanced attentional capture by salient aversive events due to a loss of this active top down attentional mechanism.

Besides a marked increase in emotional reactivity to predator threat, lesions of either the antOFC or vlPFC also significantly attenuated “coping strategy” responses. This effect was mainly driven by reduced numbers of tsik vocalizations, the mobbing and alarm call made to a threatening stimulus (Bezerra and Souto, 2008; Clara et al., 2008). Based on its association with a change in transient cortisol levels, this call has been regarded as part of a coping response in stressful situations (Cross and Rogers, 2006; Clara et al., 2008). It has been reported in both captive and wild marmosets (Barros et al., 2002; Bezerra and Souto, 2008), and was emitted in large numbers in our sham-operated controls. The lesion-induced *reduction* in response to predator threat should be contrasted with the marked *increase* in tsik and tsik-egg calls made by these same vlPFC lesioned animals, when compared to controls, in response to a human intruder (Agustín-Pavón et al., 2012), a context in which intact animals are far less likely to make tsik calls. A reduction in calls in the present study rules out a simple explanation for the increase in calls in the previous study being due to a loss of inhibitory control (Aron, 2007).

One potential explanation for the opposing effects of lesions on vocalizations in response to predator threat and a human intruder may lie in the interaction between overall levels of emotionality and the coping strategy adopted. Emotional reactivity and coping response are not necessarily independent psychological dimensions. The strength of the emotional response may interact directly with the cognitive strategy adopted and may follow an inverted U-shaped function. Hence, when emotional reactivity to a human intruder, which is normally much less than that to a snake, is increased following PFC lesions, this may increase the likelihood that an animal adopts an active/aggressive strategy. In contrast, when emotional reactivity to a snake is increased following PFC lesions, the overall level of reactivity may be considerably greater, such that it acts to reduce the likelihood of an animal adopting an active/aggressive strategy and instead induces a withdrawal response, including an inhibition of vocalizations. In support of this, those animals in the colony that show the most extreme withdrawal response to the snake tend to also stay silent.

However, an alternative and equally plausible explanation is that the lesions disrupted the decision making process *per se*. Given that marmosets display distinct patterns of behavior, including distinct vocalizations, in response to a human intruder and snake, then it is essential that animals recognize the different social and biologically relevant stimuli, and implement the appropriate coping behaviors. A snake commonly predates on marmoset monkeys in the wild (Ferrari and Ferrari, 1990; Correa and Coutinho, 1997; Ferrari and Beltrão-Mendes, 2011) and is regarded as an evolutionary relevant fear stimulus in primates (Öhman and Mineka, 2001; Mineka and Öhman, 2002; Isbell, 2006), whereas an unfamiliar human, which is not a natural predator of marmosets, can be seen as a more ambiguous and potentially dangerous social stimulus (Rudebeck et al., 2006; Machado et al., 2009). The vlPFC receives processed information of stimuli's visual characteristics from the inferior temporal cortex (Kringelbach and Rolls, 2004), is involved in guiding the selection and retrieval of semantic knowledge of the stimulus (O'Reilly, 2010), is activated by social judgments (Farrow et al., 2011) and its white matter volume is negatively correlated with social deficits in autistic children (Girgis et al., 2007). Thus, the vlPFC may be in a position to influence and regulate the implementation of appropriate coping behaviors such as proactive aggression (Blair, 2003, 2004). Without a vlPFC animals may show a general impairment in implementing the appropriate stimulus-specific and context-dependent strategy.

In conclusion, the present study demonstrates that localized excitotoxic lesions of either the primate antOFC or vlPFC leads to enhanced fear-related responses to a predator threat, which implicates these ventral prefrontal sub-regions, not only in the regulation of conditioned fear and anxiety, as we had shown previously (Agustín-Pavón et al., 2012), but also innate threat. Moreover, lesions of either region reduced the likelihood of animals adopting an active coping strategy, but whether this effect was an indirect result of the overall increase in their sensitivity to threat, leading to withdrawal, or a direct effect on decision making *per se*, remains to be determined. The finding that the pattern of emotion dysregulation appears similar following lesions of these two anatomically distinct regions leaves open the question as to their differential contributions. Given that activity in OFC neurons codes for upcoming appetitive and aversive motivational outcomes (Murray et al., 2007; Salzman and Paton, 2007; Schoenbaum et al., 2009), the lesion-induced loss of this coding would be expected to increase overall uncertainty in an animal's environment, a major contributor for heightened anxiety (Grupe and Nitschke, 2013) and may thus explain the heightened responsivity of the OFC lesioned marmosets to the model snake, compared to controls. This may have been particularly apparent when encountering the snake in what is normally the relatively safe environment of their home cage, since controls would presumably have been able to use this knowledge to regulate their emotional responses accordingly, whereas the loss of predictability in the antOFC lesioned animals would lead to excessive fear responses and withdrawal. On the other hand, the vlPFC has been implicated in top down attentional control and cognitive reappraisal of negative stimuli (Ochsner et al., 2002; Phan et al., 2005). Thus, whether in response to updated contextual information received from the OFC, the vlPFC inhibits attentional capture by the salient aversive stimulus, facilitating reappraisal of the biological and social relevance of the confronting stimulus, leading to situation-relevant emotional and coping responses, needs further investigation. However, the present results do highlight how dysregulation in distinct prefrontal regions can lead to an apparently similar behavioral phenotype, in this case, heightened emotionality, a core symptom of many neuropsychiatric disorders, including the mood and anxiety disorders. By dissecting out each region's independent contribution, we will begin to provide insight into the varied etiology of these disorders, allowing for more precise diagnostics and better targeting of treatments.

### Conflict of interest statement

The authors declare that the research was conducted in the absence of any commercial or financial relationships that could be construed as a potential conflict of interest.
